# Comparing the Efficacy and Safety of Obeticholic Acid and Semaglutide in Patients With Non-Alcoholic Fatty Liver Disease: A Systematic Review

**DOI:** 10.7759/cureus.24829

**Published:** 2022-05-08

**Authors:** Nabeel R Ahmed, Vaishnavi Vijaya Kulkarni, Sushil Pokhrel, Hamna Akram, Arowa Abdelgadir, Abanti Chatterjee, Safeera Khan

**Affiliations:** 1 Clinical Research, California Institute of Behavioral Neurosciences & Psychology, Fairfield, USA; 2 Medicine, Dow International Medical College, Karachi, PAK

**Keywords:** non-alcoholic steatohepatitis, non-alcoholic fatty liver disease, obeticholic acid, semaglutide, liver fibrosis, chenodeoxycholic acid, glucagon-like peptides

## Abstract

Patients with non-alcoholic fatty liver disease (NAFLD) have an increased risk of developing progressive fibrosis, cirrhosis, and hepatocellular carcinoma. As of now, there are no FDA-approved treatments for NAFLD/non-alcoholic steatohepatitis (NASH) or its associated fibrosis. Although many drugs are under clinical trial, both obeticholic acid (OCA) and semaglutide are among the few that have reached phase III clinical trials, but they were never compared.

We decided to conduct a systematic review of randomized controlled trials and meta-analyses. A total of 6,589 articles were found after searching PubMed, OVID Embase, OVID Medline, PubMed Central, and clinicaltrials.gov. Only full-text peer-reviewed articles published in the past six years were put through the Cochrane bias assessment tool or the Assessment of Multiple Systematic Reviews (AMSTAR) tool to screen for bias. After strict quality assessment, data from five randomized controlled trials (n=2,694) and three systematic reviews/meta-analysis (n=8,898) was extracted and included.

The data extraction from these studies showed that semaglutide and OCA cause histological improvement, but NASH resolution is exclusive to semaglutide. Although high doses of OCA can cause dyslipidemia and severe pruritus, it is the only therapeutic that causes improvement in NASH-associated hepatic fibrosis. Semaglutide is the safest option among the two and leads to significant weight loss compared to OCA; thus, a better outcome on hepatic steatosis follows. The indications of each of these drugs should be based on the NAFLD activity score and NASH fibrosis stage. OCA should be used with caution among patients with hyperlipidemia and ischemic heart disease as it may make these conditions worst.

## Introduction and background

According to a Markov model, in the USA, predictions show that non-alcoholic fatty liver disease (NAFLD) will have a 21% increase from the years 2015 to 2030, leading to a 33.5% presence by 2030 [1]. By the year 2030, this 63% surge in non-alcoholic steatohepatitis (NASH) cases will lead to a 168% surge in patients with decompensated cirrhosis and a 137% surge in patients developing hepatocellular carcinoma [1].

**Figure 1:**
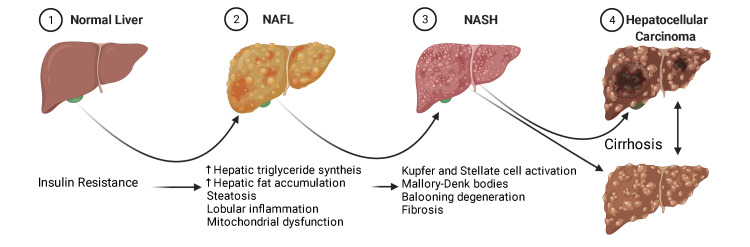
NAFLD progression sequence NAFL, non-alcoholic fatty liver; NASH: non-alcoholic steatohepatitis The figure is authors’ original illustration

Steatohepatitis and fatty liver disease can have various etiologies, but to be classified as NAFLD, other causes of steatosis must be excluded, such as alcohol consumption [2]. NAFLD is characterized by hepatic steatosis or buildup of fat in the liver [2]. NASH is an advanced stage of NAFLD characterized by steatosis and findings of liver cell injury with inflammation leading to fibrosis [2]. The early stage of NAFLD is non-alcoholic fatty liver, which is generally a benign, non-progressive disease [2]. The hepatocellular injury in NASH is caused by an increase in metabolic substrates (glucose, fructose, and fatty acids), leading the fatty acids to participate in pathways that cause cellular injury and a poor response to that injury [3]. The pathogenesis of this disease is largely associated with obesity, type 2 diabetes, and increasing age [3]. NAFLD has been projected to become the most common cause of liver transplantation within a decade [3]. We created Figure 1 in order to summarize the progression of NAFLD and its related features.

Multiple clinical trials are in progress and have been conducted to find an approved medical treatment of NAFLD/NASH [4]. Currently, no FDA-approved medications are listed for NAFLD or NASH and its associated fibrosis [5]. Lifestyle changes are recommended, with studies showing that losing around 10% of body weight may lead to resolution and regression of NASH [6]. Among all the drugs being assessed for the treatment of NAFLD, no single one has shown the right combination of effects and safety required to be considered for FDA approval [7].

Semaglutide, a GLP-1 (glucagon-like peptide 1) agonist, is a well-researched FDA-approved drug for type 2 diabetes in 2019 and chronic weight management in 2021. Semaglutide has become a drug of interest for NASH since many studies show a decrease in alanine transaminase (ALT) and high-sensitivity C-reactive protein (hs-CRP) in patients diagnosed with type 2 diabetes and obesity [8]. Obeticholic acid (OCA) is a semi-synthetic analog derived from chenodeoxycholic acid, a natural ligand for the Farnesoid X receptor (FXR) [9]. OCA causes a decrease in inflammatory cell infiltration and fibrosis due to the reduction in hepatic monocyte chemoattractant protein-1 (MCP-1) mRNA [10]. OCA is approved by the FDA for the treatment of primary biliary cholangitis. Due to its anti-fibrotic, anti-cholestatic, and anti-inflammatory properties, OCA is currently under a six-year-long phase III clinical trial regarding its therapeutic effects in NASH [10]. Both of these drugs have gone through extensive trials and analyses in regard to NAFLD. Although lifestyle changes are critical, it is not always possible, making additional therapeutic agents necessary to combat this upcoming pandemic of NAFLD. Due to the lack of any FDA-approved treatment, we decided to perform a systematic review to assess the safety and efficacy of semaglutide and OCA in patients with NAFLD to reduce liver fibrosis and resolution.

## Review

Methods

**Figure 2:**
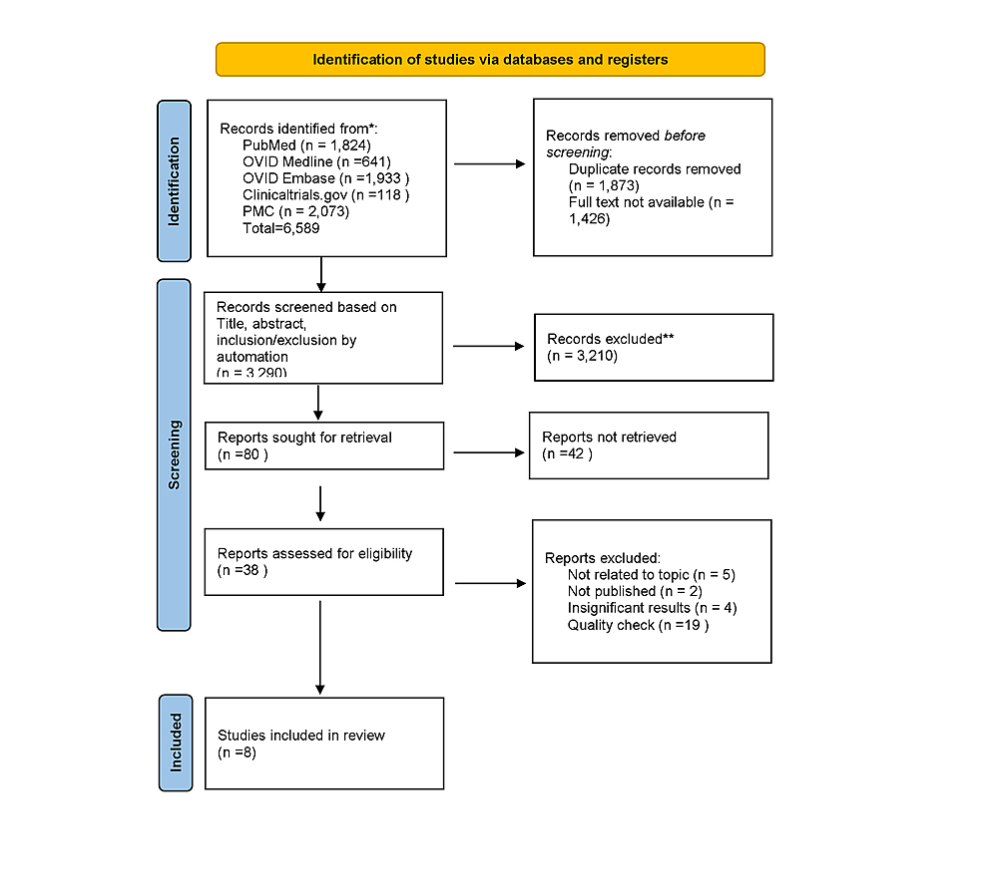
PRISMA 2020 flow chart PRISMA, Preferred Reporting Items for Systematic Reviews and Meta-Analyses

The Preferred Reporting Items for Systematic Reviews and Meta-Analyses (PRISMA) 2020 guidelines and principles were used to design this systematic review and report its results, with a full breakdown shown in Figure 2 [11].

Search strategy

We used major literature databases, including PubMed, OVID Embase, OVID Medline, PubMed Central, and clinicaltrials.gov. When searching, we used appropriate keywords and the Medical Subject Headings (MeSH) thesaurus to find relevant articles mentioning OCA or semaglutide's efficacy and safety in treating NASH.

For our literature search, the keywords used were "Obeticholic acid," "Semaglutide," "Non-alcoholic fatty liver disease," "Liver Fibrosis," "GLP-1 agonist". Using PubMed Central, we searched for their corresponding MeSH terms. The combined MeSH terms for all of the aforementioned keywords are as follows: Non-alcoholic fatty liver disease OR NAFL OR NAFLD OR metabolic associated fatty liver disease OR MAFLD OR non-alcoholic steatohepatitis OR NASH OR ( "Non-alcoholic Fatty Liver Disease/drug therapy"[Majr] OR "Non-alcoholic Fatty Liver Disease/prevention and control"[Majr] ) AND Obeticholic acid OR Ocaliva OR ( "Chenodeoxycholic Acid/administration and dosage"[Majr] OR "Chenodeoxycholic Acid/adverse effects"[Majr] OR "Chenodeoxycholic Acid/therapeutic use"[Majr] OR "Chenodeoxycholic Acid/toxicity"[Majr] ) AND GLP-1 agonist OR Ozempic OR Semaglutide OR ( "Glucagon-Like Peptides/administration and dosage"[Majr] OR "Glucagon-Like Peptides/adverse effects"[Majr] OR "Glucagon-Like Peptides/therapeutic use"[Majr] OR "Glucagon-Like Peptides/toxicity"[Majr] ).

For OVID Medline and OVID Embase, we used the following keywords combined with Booleans: Non-alcoholic fatty liver disease OR NAFL OR NAFLD OR metabolic associated fatty liver disease OR MAFLD OR non-alcoholic steatohepatitis OR NASH AND Obeticholic acid OR Ocaliva OR Farnesoid X Receptor Agonists AND GLP-1 agonist OR Ozempic OR Semaglutide. For clinicaltrials.gov, we used "NAFLD."

After compiling the papers using the search strategy, we screened each paper. We removed duplicates, went through the title and abstracts, and only selected full-text papers after applying the detailed eligibility criteria. If the full-text article was not extracted, the paper was removed.

Inclusion and exclusion criteria

We selected articles published within the past six years (2015-2021) in English. We filtered to include only peer-reviewed randomized controlled trials (RCTs) and meta-analysis to have the most accurate and up-to-date information. Grey literature was not used, and only full-text articles were considered.

Analysis of quality of each study and data extraction

Each paper was screened using a quality appraisal tool. The systematic reviews/meta-analyses were screened using the Assessment of Multiple Systematic Reviews (AMSTAR) tool (Table 1).

**Table 1:** AMSTAR tool AMSTAR, Assessment of Multiple Systematic Reviews

AMSTAR Criteria (Yes, No, Uncertain)	Kulkarni et al., 2021 [10]	Majzoub et al., 2021 [7]	Mantovani et al., 2021 [12]
A priori design	Uncertain	Uncertain	Uncertain
Duplicate study selection and data extraction	Uncertain	Yes	Uncertain
Literature search	Yes	Yes	Yes
Status of publication	Yes	Yes	Yes
List of studies	Yes	Yes	Yes
Characteristics of included studies	Yes	Yes	Yes
Scientific quality	Yes	Yes	Yes
Formulation of conclusion	Yes	Yes	Yes
Method used to combine findings	Yes	Yes	Yes
Likelihood of publication bias	Yes	Yes	Yes
Conflict of interest	Yes	Yes	Yes
Our evaluation	9/11 (medium quality)	10/11 (high quality)	9/11 (medium quality)

The RCTs were screened using the Cochrane bias assessment tool (Table 2).

**Table 2:** Cochrane bias assessment tool

Cochrane Criteria (Yes, No, Uncertain)	Baekdal et al., 2018 [13]	Flint et al., 2021 [14]	Neuschwander-Tetri et al., 2015 [15]	Newsome et al., 2021 [16]	Younossi et al., 2019 [17]
Adequate sequence generation?	Yes	Yes	Yes	Yes	Yes
Allocation concealment used?	Uncertain	Uncertain	Uncertain	Yes	Uncertain
Blinding?	Yes	Yes	Yes	Yes	Yes
Are concurrent therapies similar?	No	Yes	Yes	Yes	Yes
Incomplete outcome data addressed?	Yes	Yes	Yes	Yes	Yes
Uniform and explicit outcome definitions?	Yes	Yes	Uncertain	Yes	Yes
Free of selective outcome reporting?	Yes	Yes	Yes	Yes	Yes
Free of other bias?	Yes	Yes	Yes	Yes	Yes
Overall risk of bias?	Yes	Yes	Yes	Yes	Yes
Our Evaluation	7/9 (medium quality)	8/9 (high quality)	8/9 (high quality)	9/9 (high quality)	8/9 (high quality)

The papers were then rated from a range of high, medium, or low quality. Only the papers that were rated as medium or high quality were used. The other medium-quality papers that did not meet our quality appraisal were used in basic concept explanations of our paper. We then extracted information related to both drugs' safety and efficacy, comparing and contrasting information from each selected study.

Results

Our initial search yielded 6,589 articles. Out of these, we removed duplicates (n=1,873) and those without the full text available (n=1,426). We were left with 3,290 articles. Next, we screened the articles based on title, abstract, inclusion/exclusion criteria manually and by automation (n=3,290). This screened out 3,210 articles, leaving 80 articles that we sought for full-text retrieval. The full text of 42 of these articles was not retrieved, leaving us with 38 articles. Finally, after a thorough review using strict quality checks and inclusion criteria, we included eight articles in our systematic review.

Our systematic review contains four RCTs, one interim analysis of an ongoing phase III RCT, and three systematic reviews with meta-analysis that include either semaglutide or OCA's efficacy or safety in treating patients with NAFLD or hepatic impairment. All the analyzed articles have a population of confirmed NASH/NAFLD patients except one of the RCTs examining the safety of semaglutide in patients with hepatic impairment. Refer to Table 3 for the individual study breakdown.

**Table 3:** Breakdown of individual studies included in the review AE, adverse effects; ALT, alanine transaminase; AST, aspartate aminotransferase; BMI, body mass index; BP, blood pressure; CTP, Child-Turcotte-Pugh classification; ELF, enhanced liver fibrosis; GI, gastrointestinal; GLP-1, glucagon-like peptide 1; HDL, high-density lipoprotein; LDL, low-density lipoprotein; MRI, magnetic resonance imaging; MRS, magnetic resonance spectroscopy; NAFLD, non-alcoholic fatty liver disease; NASH, non-alcoholic steatohepatitis; OCA, obeticholic acid; RCT, randomized controlled trial Obese: BMI ≥ 30 kg/m^2^

Study/Year	Location	Study Type	Drugs Used/Patient Group	Result	Conclusion	Total Patient Population/Comorbidities
Kulkarni et al., 2021 [10]	India	Systematic review, meta-analysis	OCA in patients w/ NASH	25 mg and 10 mg of OCA showed histological improvement. Increased the risk of pruritus mainly from 25-mg dose. No steatosis improvement was shown. Improved ELF score, thus improving fibrosis.	25 mg of OCA may be more potent and effective for NASH resolution, but 10 mg of OCA is the adequate alternative due to AEs.	2,834
Majzoub et al., 2021 [7]	USA	Systematic review, meta-analysis	OCA, pioglitazone, semaglutide, liraglutide in patients w/ NASH	RCTs show that OCA was superior to placebo in ≥ one stage improvement in fibrosis. Network meta-analysis showed that semaglutide was ranked the most effective for NASH resolution	Therapies that improve NASH resolution be combined with therapies that have an anti-fibrotic effect should be assessed.	5,129
Mantovani et al., 2021 [12]	Italy	Systematic review, a meta-analysis	GLP-1 receptor agonists in patients w/ NAFLD or NASH	No significant AE. Increased frequency of GI symptoms. Decreased liver fat content assessed using MRI or MRS was up to 32%. Significant improvement of hepatic steatosis. Semaglutide showed histological resolution of NASH with no worsening fibrosis.	MRI and liver histology proves that GLP-1 receptor agonist agonists improve NAFLD. If confirmed through larger phase III RCTs with liver biopsy, therapy should be considered.	935
Baekdal et al., 2018 [13]	Denmark	RCT	Oral semaglutide in patients w/ hepatic impairment and w/o hepatic impairment	Headache was the most frequently reported AE (14.3%) along with GI symptoms: hypoglycemic episodes also occurred w/ glucose level of 70 mg/dL reported in a few patients.	Patients tolerated oral semaglutide well. AEs are not significant.	56 patients total. 6 patients w/ type 2 diabetes, 12 in CTP class A, 12 in CTP class B, and 8 in CTP class C
Flint et al., 2021 [14]	Denmark	Phase I RCT	Subcutaneous semaglutide 0.4 mg in patients w/ NAFLD	Reductions in liver steatosis were significantly greater with semaglutide at weeks 24, 48, and 72. Decreased liver enzymes, body weight, and HbA1c. Decreased appetite and nausea were reported.	Didn't have a significant impact on liver stiffness. Decreased steatosis, along with w/ decreased liver enzymes and metabolic parameters, shows a good impact on disease activity.	67 total. 48 patients w/ type 2 diabetes and 62 patients classified obese
Neuschwander-Tetri et al., 2015 [15]	USA	Phase IIb RCT	OCA in patients ≥ 18 years old. Liver biopsy proven NASH or borderline NASH, NAFLD activity score ≥ 4.	45% of patients improved liver histology. The resolution was the same with a placebo. Increased serum cholesterol and LDL w/ decrease in HDL and decrease in serum ALT and AST. Weight loss and decreased systolic BP. AE: pruritus, hyperglycemia, dysarthria, dizziness, and insulin resistance	OCA improves histological features of NASH, but long-term safety requires further investigation. May increase risk of atherogenesis. More trials are needed on the resolution of NASH from OCA.	283 total. 149 patients w/ type 2 diabetes and 173 patients w/ hyperlipidemia
Newsome et al., 2021 [16]	UK	Phase II RCT	Subcutaneous semaglutide patients with biopsy-confirmed NASH and liver fibrosis of stage F1, F2, or F3	40% of patients achieved NASH resolution without worsening of fibrosis. Improvement in fibrosis stage occurred in 43%, 13% mean weight loss occurred in those receiving 0.4 mg. GI disturbances were higher in 0.4 mg Malignant neoplasms were reported in three patients.	Semaglutide causes NASH resolution. Improvement in fibrosis is not substantial since the placebo group also improved.	320 total. 199 patients w/ type 2 diabetes. Mean BMI: 35.8.
Younossi et al., 2019 [17]	USA	Ongoing RCT phase III interim analysis	OCA 10 mg or OCA 25 mg in patients w/ definite NASH. NAFLD activity score of ≥4 and liver fibrosis of stage F2/F3.	OCA 25 mg significantly improved fibrosis. Clinically significant histological improvement was noted.	Likely to predict clinical benefit. Indicated for patients with advanced fibrosis.	931 total. 517 patients w/ type 2 diabetes. 633 patients w/ dyslipidemia.

Both OCA and semaglutide effectively improve histology of the liver as assessed by the NAFLD activity score (NAS) [10,12]. Semaglutide achieved NASH resolution without worsening fibrosis, while OCA failed to do so [15,16]. OCA is highly effective in improving liver fibrosis caused by NASH, whereas semaglutide is only effective in halting fibrosis progression [10,17]. Semaglutide achieved greater weight loss than OCA without a significant rebound weight gain after drug cessation [12,15-17]. The adverse effects (AEs) of semaglutide are non-significant at any dose, with headache and loss of appetite being the most frequently reported AE followed by gastrointestinal (GI) symptoms: dyspepsia, vomiting, decreased appetite, and diarrhea [13]. OCA comes with its own set of AE, making its use rather controversial. These can include severe pruritus, hyperglycemia, dysarthria, dizziness, and insulin resistance [15]. One of the most significant AE is decreased hepatic lipogenesis, leading to increased total cholesterol and low-density lipoprotein (LDL) [15].

Discussion

Both semaglutide and OCA are among the few promising drugs that have the potential of becoming FDA-approved for NAFLD therapy. This is the first systematic review to compare the efficacy and safety of semaglutide and OCA regarding NAFLD to the best of our knowledge. NAFLD/NASH has become the most common etiology of chronic liver disease (CLD) worldwide [18]. The development of cirrhosis (due to fibrosis) is predictive of a poor prognosis of liver-related morbidity and mortality [18,19]. The prevention or decrease in fibrosis may keep the patient from developing cirrhosis and CLD [19]. Some risk factors of NASH are controllable, including obesity and type 2 diabetes, making lifestyle changes a viable option. The same cannot be said about patients with uncontrollable risk factors and those who are unable to make the lifestyle changes required.

Mechanism of Action of Semaglutide and Obeticholic Acid

NAFLD is caused by an increased accumulation of lipids, leading to hepatic lipotoxicity. In patients with insulin resistance, most of the free fatty acid (FFA) pool comes from adipose tissue lipolysis along with minor amounts from the diet or lipogenesis. These FFAs will be oxidized and become triglycerides, and this increased accumulation may lead to steatosis or get secreted as very LDL [19]. Hepatic stellate cells help mediate the fibrosis associated with NASH, causing type I collagen and connective tissue growth factor expression [20]. In patients with excessive abdominal adipose tissue, there is an increased production and release of inflammatory cytokines such as tumor necrosis factor alpha and interleukin-6, resulting in a cycle of inflammation and toxicity, leading to further fibrosis [20].

Semaglutide is a GLP-1 receptor agonist, primarily used for type 2 diabetes and weight loss [21]. It activates the peroxisome proliferator-activated receptor (PPAR-α) of the liver, which decreases the production of apolipoprotein-C and breaks down fat in plasma and triglycerides [20,21]. The activation of this receptor may also cause delayed gastric emptying. Overall, this improves lipid metabolism and prolongs the feeling of satiety, decreasing waist circumference. The waist circumference is directly proportional to insulin resistance. The pancreatic islet β and Δ cells express GLP-1 receptors to control insulin [21]. Therefore, semaglutide causes an increase in insulin production and secretion with a decrease in glucagon secretion.

Semaglutide is administered subcutaneously at doses of 0.1 mg, 0.2 mg, or 0.4 mg for NAFLD/NASH. It can also be taken orally but is not as effective. According to a study, the oral form of semaglutide was not as effective as 0.5 mg and 1.0 mg of subcutaneous semaglutide in reducing weight [22].

OCA is a semi-synthetic, modified bile acid derived from chenodeoxycholic acid [23]. OCA is primarily used for the treatment of primary biliary cholangitis. It is a highly selective agonist of the FXR found in the liver, kidney, adrenal glands, and intestines [24]. Lipophilic bile acids have been known to regulate metabolism and insulin sensitivity. When lipophilic bile acids are attached to the FXR, insulin sensitivity is increased along with a decrease in hepatic gluconeogenesis and serum triglycerides [15]. FXR activation causes expression of the hepatic scavenger receptors (SRB1), which increases HDL clearance [15]. The mechanism of OCA decreasing hepatic fibrosis in humans is unclear. FXR's agonists regulate the production of bile acids by causing the release of fibroblast growth factor-19, which suppresses cholesterol 7-alpha-hydroxylase (CYP7A1), preventing cholesterol conversion to bile acid [25]. This can cause unwanted AEs such as hyperlipidemia and an increase in LDL.

Comparing Both Semaglutide and Obeticholic acids Efficacy on NAFLD/NASH Patients

**Figure 3:**
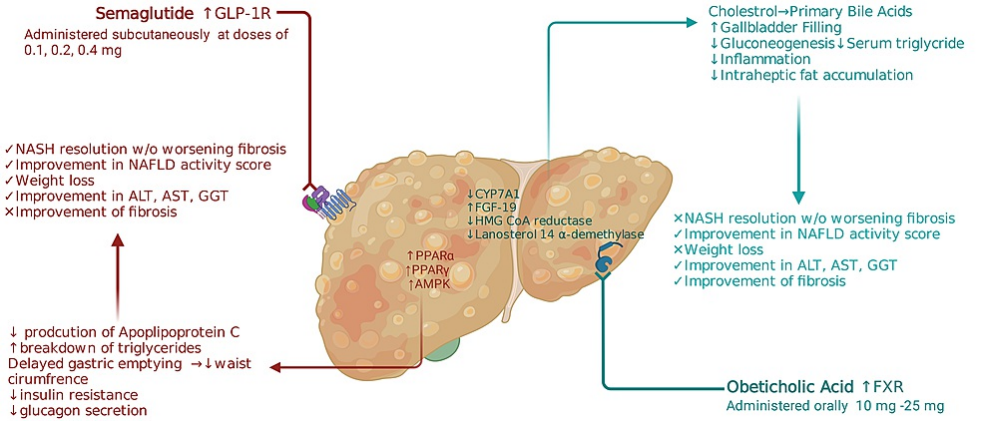
Mechanism of action and effects on NAFLD/NASH ALT, alanine aminotransferase; AMPK, activated protein kinase; AST, aspartate aminotransferase; CYP7A1, cholesterol 7 alpha-hydroxylase; FXR, Farnesoid X receptor; FGF-19, fibroblast growth factor 19; GGT, gamma-glutamyl transpeptidase; GLP-1R, glucagon-like peptide 1 receptor; NAFLD, nonalcoholic fatty liver disease; NASH, nonalcoholic steatohepatitis; PPAR, peroxisome proliferator-activated receptor Authors’ original illustration

Many therapeutic categories are sought out in regard to NASH. These can include NASH resolution without worsening fibrosis, decrease in METAVIR fibrosis stage (liver stiffness), decrease in NAS (histological improvement), and weight loss. We created Figure 3 to outline the mechanism of action and effects of both drugs. We broke down each of these categories and compared each drug based on the selected studies.

NASH resolution without worsening of fibrosis

Resolution of NASH entails histological absence of hepatocyte ballooning with decreased or absence of inflammation [26]. OCA was assessed in a multicenter phase IIb RCT of 283 patients compared to a placebo group [15]. There was a lack of resolution of NASH with similar rates as the placebo group. But according to Younossi et al.'s interim analysis at 18 months, OCA 25 mg achieved NASH resolution twice as much as the placebo group [17]. Although still ongoing, we believe that the phase III RCT interim analysis findings should be taken into consideration since it was a longer study and more recent. The difference in results may be due to bias in a pathologist post hoc analysis conducted in the ongoing phase III RCT.

In 2021, Newsome et al. conducted a phase II double-blind RCT in biopsy-confirmed NASH patients comparing the efficacy of subcutaneous semaglutide 0.1, 0.2, and 0.4 mg with a placebo group. Among patients with F2/F3 fibrosis, NASH resolution without worsening fibrosis was highest in the 0.4 mg group [16]. According to a recent meta-analysis, both liraglutide and semaglutide showed evidence of NASH resolution [12]. In Majzoub et al.’s network meta-analysis forest plot, both OCA and semaglutide were given a SUCRA (surface under the cumulative ranking curve) score in regard to their efficacy in resolution of NASH. Among 20 other possible therapeutics, subcutaneous semaglutide 0.4 mg was given the highest SUCRA score of 89%, whereas OCA was only about half of that [7]. Semaglutide is the most effective for NASH resolution, but there is a lack of concrete evidence that OCA leads to NASH resolution.

Fibrosis stage and liver stiffness

Fibrosis improvement is a crucial part of NASH therapy. Magnetic resonance imaging proton density fat fraction (MRI-PDFF) and magnetic resonance elastography (MRE) are two imaging techniques that assessed liver stiffness and steatosis in Flint et al.’s study on the effects of semaglutide [14]. The stages can vary from stage 0-4, no fibrosis to cirrhosis, respectively [27]. According to the study, there was no significant improvement in liver stiffness compared to placebo [14]. In another RCT involving 320 patients taking subcutaneous semaglutide, groups did not significantly reduce fibrosis either [16]. Although semaglutide may not improve fibrosis, the studies consistently indicated protection against worsening fibrosis [14,16]. In both studies, majority of patients had either type 2 diabetes or obesity, with semaglutide improving both of these conditions.

OCA has been found to be the only drug to significantly impact fibrosis in multiple studies [7,10,15,17]. The phase III RCT interim analysis at 18 months that included 1,968 patients was long enough to assess this relatively slow process. In Kulkarni et al.’s meta-analysis funnel plot, 25 mg of OCA showed significantly better fibrosis improvement than the placebo group with an odds ratio of 1.95 and a small confidence interval [10]. These results are consistent with other RCTs and systematic reviews, making OCA a highly effective candidate for NASH fibrosis stage improvement. OCA 10 mg is also sufficient enough to improve fibrosis and should be the recommended dose for NASH since OCA 25 mg does substantially increase the risk of AEs.

NAFLD activity score (NAS)

The NAS helps distinguish NASH from NAFL along with any significant changes in histology [28]. Both drugs showed a significant improvement in NAS.

Studies assessing semaglutide's efficacy indicated an improvement of the NAS (≥1), with similar effects found in all doses [12,14,16]. We believe that this was due to the combined effect that GLP-1 receptor agonists have on weight loss and insulin resistance. Newsome et al.’s RCT showed that almost all patients receiving subcutaneous semaglutide had ≥1 improvement in the NAS [16].

According to two RCTs, OCA is also effective in improving histology and NAS [15,17]. These studies broke down each category of the NAS and the histological improvement associated. It is rather odd that OCA has shown an improvement in NAS but failed to reach NASH resolution. We believe that NAS is not a good indicator for NASH resolution and rather a scale of severity of the disease.

Weight loss

The only FDA-approved therapy for NAFLD/NASH is lifestyle changes leading to weight loss. Studies have shown that 7-10% of weight reduction may lead to regression of fibrosis and a significant decrease in liver enzymes, liver steatosis, and fibrosis [14,29,30]. One would expect medication-induced weight loss to have a similar effect. This weight loss is not ideal because weight gain after cessation of medication is probable.

On average, GLP-1 RAs can cause 4-5 kg of weight loss and continue up to week 44 of the regimen [12,16]. These findings were unexpected since regression of fibrosis did not occur even though weight loss was significant.

OCA caused weight loss of approximately 2% and significantly rebounded after treatment discontinuation [15,17]. With obesity being one of the most common associations, OCA failed to show promising results in this essential therapy category. This amount is lower than expected when considering the decrease in liver enzymes and significant liver histology (NAS) improvement.

Adverse Effects of Obeticholic Acid and Semaglutide in Patients With NASH or Hepatic Impairment

Many clinical trials have been conducted assessing the safety of these drugs, but very few contain the long-term clinical effects of OCA on cardiovascular health. The safety and AEs of semaglutide change slightly based upon the mode of admission. OCA's AEs substantially change based on increasing dosages.

Among 56 patients with or without hepatic impairment taking oral semaglutide in a multicenter RCT, almost all AEs reported were mild or moderate [12]. According to this study, the most common AE was a headache, but in another RCT assessing subcutaneous semaglutide's safety, headache was not mentioned as a significant AE [13,16]. We believe that this difference in AE is due to the mode of administration. In all other studies regarding the safety of semaglutide, GI effects were reported as the most common AE [16]. The use of semaglutide should be monitored in patients with pre-existing GI conditions. All four of the studies on semaglutide have indicated that semaglutide is a safe well studied therapeutic for NAFLD/NASH patients [12-14,16].

The AEs of OCA are important to consider when assessing a patient for treatment. Higher doses of OCA (25-50 mg) lead to more significant AE; according to Younossi et al.’s interim analysis, OCA 25 mg led to the highest rates of pruritus and hyperlipidemia compared to OCA 10 mg [17]. We believe that both of these are class effects due to the activation of the FXR. Cilofexor is another FXR agonist with pruritus as a common AE [31]. Pruritis may be avoided to a great degree with OCA 10 mg and should be the recommended daily dosage.

It is unknown if OCA-induced hyperlipidemia is associated with cardiovascular mortality. In a phase II RCT, the addition of atorvastatin decreased the LDL and the mean LDL particle concentration levels below baseline in all dosages of OCA [32]. The use of statins along with OCA may help regulate dyslipidemia caused by OCA, making this a safer option for at-risk patients. Other AEs are not significant, and OCA is generally well-tolerated [17]. More research should be done to determine the long-term safety of OCA and its impact on at-risk cardiovascular patients.

According to multiple studies, among patients with NAFLD, approximately 51% are obese and up to 80% have dyslipidemia [33,34]. Therefore, high doses of OCA (25-50 mg) can cause dangerous AEs among the vast majority of NAFLD patients.

Limitations 

This systematic review has some potential limitations. There are currently no clinical trials directly comparing the effects and safety of semaglutide with OCA. All the RCTs we selected were assessing each drug individually. Our exclusion criteria did not allow us to use articles that did not contain an abstract, further limiting article selection. Lastly, most studies on NAFLD/NASH do not mention a definitive diagnostic method used to determine patient selection limiting quality studies.

## Conclusions

By comparing the efficacy and safety of semaglutide and OCA, it is evident that each drug is meant for its own specific patient population based on therapy goals and comorbidities. Both therapies improve liver histology and liver enzymes, but other therapeutic effects differed. Semaglutide is most effective for NASH resolution. OCA is unique because of its effects on hepatic fibrosis, which is the biggest predictor of liver mortality in NASH. We would like to see trials of both of these drugs used for their combined therapeutic effects. At high doses, OCA may induce hyperlipidemia and severe pruritus, making its use contradictory toward the risk factors of NASH. If fibrosis improvement is not the mainstay of treatment, then the use of OCA is not advised, especially among patients with risk factors of heart disease. However, our study results show that to minimize these AEs, an OCA regimen should be started at lower doses, and, if required, combining it with a statin or antipruritic. Semaglutide largely differed in this aspect, being unremarkably safe and well-studied both in the long-term and short-term safety. Lifestyle modifications will remain critical, but additional therapeutics will be necessary to combat the projected surge of NAFLD especially among patients with type 2 diabetes or obesity.

Our study consists of data from recent high-quality RCTs and meta-analyses, giving an updated evaluation of the possible future management of NAFLD. We hope this will provide clinicians and the respective regulators with the evidence necessary to move one step closer toward an approved therapeutic.

## References

[1] Estes C, Razavi H, Loomba R, Younossi Z, Sanyal AJ (2018). Modeling the epidemic of nonalcoholic fatty liver disease demonstrates an exponential increase in burden of disease. Hepatology.

[2] Wilkins T, Tadkod A, Hepburn I, Schade RR (2013). Non-alcoholic fatty liver disease: diagnosis and management. Am Fam Physician.

[3] Neuschwander-Tetri BA (2017). Non-alcoholic fatty liver disease. BMC Med.

[4] Negi CK, Babica P, Bajard L, Bienertova-Vasku J, Tarantino G (2022). Insights into the molecular targets and emerging pharmacotherapeutic interventions for nonalcoholic fatty liver disease. Metabolism.

[5] Chalasani N, Younossi Z, Lavine JE (2018). The diagnosis and management of nonalcoholic fatty liver disease: practice guidance from the American Association for the Study of Liver Diseases. Hepatology.

[6] Kwak MS, Kim D (2018). Non-alcoholic fatty liver disease and lifestyle modifications, focusing on physical activity. Korean J Intern Med.

[7] Majzoub AM, Nayfeh T, Barnard A (2021). Systematic review with network meta-analysis: comparative efficacy of pharmacologic therapies for fibrosis improvement and resolution of NASH. Aliment Pharmacol Ther.

[8] Newsome P, Francque S, Harrison S (2019). Effect of semaglutide on liver enzymes and markers of inflammation in subjects with type 2 diabetes and/or obesity. Aliment Pharmacol Ther.

[9] Smith SM, Pegram AH (2017). Obeticholic acid: a Farnesoid X receptor agonist for Primary biliary cholangitis. J Pharm Technol.

[10] Kulkarni AV, Tevethia HV, Arab JP (2021). Efficacy and safety of obeticholic acid in liver disease-a systematic review and meta-analysis. Clin Res Hepatol Gastroenterol.

[11] Page MJ, McKenzie JE, Bossuyt PM (2021). The PRISMA 2020 statement: an updated guideline for reporting systematic reviews. BMJ.

[12] Mantovani A, Petracca G, Beatrice G, Csermely A, Lonardo A, Targher G (2021). Glucagon-like peptide-1 receptor agonists for treatment of nonalcoholic fatty liver disease and nonalcoholic steatohepatitis: an updated meta-analysis of randomized controlled trials. Metabolites.

[13] Baekdal TA, Thomsen M, Kupčová V, Hansen CW, Anderson TW (2018). Pharmacokinetics, safety, and tolerability of oral semaglutide in subjects with hepatic impairment. J Clin Pharmacol.

[14] Flint A, Andersen G, Hockings P (2021). Randomised clinical trial: semaglutide versus placebo reduced liver steatosis but not liver stiffness in subjects with non-alcoholic fatty liver disease assessed by magnetic resonance imaging. Aliment Pharmacol Ther.

[15] Neuschwander-Tetri BA, Loomba R, Sanyal AJ (2015). Farnesoid X nuclear receptor ligand obeticholic acid for non-cirrhotic, non-alcoholic steatohepatitis (FLINT): a multicentre, randomised, placebo-controlled trial. Lancet.

[16] Newsome PN, Buchholtz K, Cusi K (2021). A placebo-controlled trial of subcutaneous semaglutide in nonalcoholic steatohepatitis. N Engl J Med.

[17] Younossi ZM, Ratziu V, Loomba R (2019). Obeticholic acid for the treatment of non-alcoholic steatohepatitis: interim analysis from a multicentre, randomised, placebo-controlled phase 3 trial. The Lancet.

[18] Li B, Zhang C, Zhan YT (2018). Nonalcoholic fatty liver disease cirrhosis: a review of its epidemiology, risk factors, clinical presentation, diagnosis, management, and prognosis. Can J Gastroenterol Hepatol.

[19] Marra F, Lotersztajn S (2013). Pathophysiology of NASH: perspectives for a targeted treatment. Curr Pharm Des.

[20] Wang XC, Gusdon AM, Liu H, Qu S (2014). Effects of glucagon-like peptide-1 receptor agonists on non-alcoholic fatty liver disease and inflammation. World J Gastroenterol.

[21] Drucker DJ (2018). Mechanisms of action and therapeutic application of glucagon-like peptide-1. Cell Metab.

[22] Meier JJ (2021). Efficacy of semaglutide in a subcutaneous and an oral formulation. Front Endocrinol (Lausanne).

[23] Chapman RW, Lynch KD (2020). Obeticholic acid-a new therapy in PBC and NASH. Br Med Bull.

[24] Abenavoli L, Falalyeyeva T, Boccuto L, Tsyryuk O, Kobyliak N (2018). Obeticholic acid: a new era in the treatment of nonalcoholic fatty liver disease. Pharmaceuticals (Basel).

[25] Novotny K, Hapshy V, Nguyen H (2021). Obeticholic acid. StatPearls [Internet].

[26] Filozof C, Chow SC, Dimick-Santos L, Chen YF, Williams RN, Goldstein BJ, Sanyal A (2017). Clinical endpoints and adaptive clinical trials in precirrhotic nonalcoholic steatohepatitis: facilitating development approaches for an emerging epidemic. Hepatol Commun.

[27] Younossi ZM, Loomba R, Anstee QM (2018). Diagnostic modalities for nonalcoholic fatty liver disease, nonalcoholic steatohepatitis, and associated fibrosis. Hepatology.

[28] de Alwis NMW (2018). Chapter 9: Obesity and non-alcoholic fatty liver disease. Practical Guide to Obesity Medicine.

[29] Hannah WN Jr, Harrison SA (2016). Effect of weight loss, diet, exercise, and bariatric surgery on nonalcoholic fatty liver disease. Clin Liver Dis.

[30] Gasteyger C, Larsen TM, Vercruysse F, Astrup A (2008). Effect of a dietary-induced weight loss on liver enzymes in obese subjects. Am J Clin Nutr.

[31] Trauner M, Gulamhusein A, Hameed B (2019). The nonsteroidal Farnesoid X Receptor agonist cilofexor (GS-9674) improves markers of cholestasis and liver injury in patients with primary sclerosing cholangitis. Hepatology.

[32] Pockros PJ, Fuchs M, Freilich B (2019). CONTROL: A randomized phase 2 study of obeticholic acid and atorvastatin on lipoproteins in nonalcoholic steatohepatitis patients. Liver Int.

[33] Godoy-Matos AF, Silva Júnior WS, Valerio CM (2020). NAFLD as a continuum: from obesity to metabolic syndrome and diabetes. Diabetol Metab Syndr.

[34] Zhang QQ, Lu LG (2015). Nonalcoholic fatty liver disease: dyslipidemia, risk for cardiovascular complications, and treatment strategy. J Clin Transl Hepatol.

